# The distribution of eukaryotic initiation factor 4E after bouts of resistance exercise is altered by shortening of recovery periods

**DOI:** 10.1186/s12576-020-00781-y

**Published:** 2020-11-04

**Authors:** Junya Takegaki, Riki Ogasawara, Karina Kouzaki, Satoshi Fujita, Koichi Nakazato, Naokata Ishii

**Affiliations:** 1 Department of Life Sciences, Graduate School of Arts and Sciences, The University of Tokyo, Tokyo, Japan; 2 Ritsumeikan Global Innovation Research Organization, Ritsumeikan University, 1-1-1 Noji-higashi, Kusatsu, Shiga 525-8577 Japan; 3 Department of Life Science and Applied Chemistry, Nagoya Institute of Technology, Nagoya, Japan; 4 Graduate School of Health and Sport Science, Nippon Sport Science University, Tokyo, Japan; 5 Faculty of Sport and Health Science, Ritsumeikan University, Kusatsu, Shiga Japan

**Keywords:** Resistance exercise, Recovery, Skeletal muscle, Protein synthesis, Translation initiation complex

## Abstract

Insufficient duration of recovery between resistance exercise bouts reduces the effects of exercise training, but the influence on muscle anabolic responses is not fully understood. Here, we investigated the changes in the distribution of eukaryotic initiation factor (eIF) 4E, a key regulator of translation initiation, and related factors in mouse skeletal muscle after three successive bouts of resistance exercise with three durations of recovery periods (72 h: conventional, 24 h: shorter, and 8 h: excessively shorter). Bouts of resistance exercise dissociated eIF4E from eIF4E binding protein 1, with the magnitude increasing with shorter recovery. Whereas bouts of resistance exercise with 72 h recovery increased the association of eIF4E and eIF4G, those with shorter recovery did not. Similar results were observed in muscle protein synthesis. These results suggest that insufficient recovery inhibited the association of eIF4E and eIF4G, which might cause attenuation of protein synthesis activation after bouts of resistance exercise.

## Background

Skeletal muscle mass is regulated by muscle protein metabolism, and the accumulation of protein synthesis causes muscle hypertrophy. Resistance exercise is well known to transiently activate protein synthesis, and resistance exercise training promotes muscle growth. The recovery period is one of the determinants of the training effect, and a period of 2 or 3 days is regarded as adequate for inducing muscle hypertrophy [1]. Resistance exercise training with an excessively short recovery period can lead to inadequate responses, such as suppression of protein synthesis and activation of muscle protein degradation systems [2–4]. However, little information is available on the mechanisms underlying the suppression of acute and chronic responses of muscle anabolism after bouts of resistance exercise training with insufficient recovery.

Although the detailed mechanisms for the activation of muscle protein synthesis and muscle hypertrophy in resistance exercise training are not fully understood, mechanistic target of rapamycin complex (mTORC) plays a role in this process [5–7]. A recent study reported that mTORC1 plays a minor role in the acute activation of muscle protein synthesis but is essential for muscle hypertrophy induced by chronic mechanical overload [8]. Additionally, repetitive resistance exercise with excessively short recovery is known to highly activate mTORC1 [2–4]. On the other hand, as mentioned above, this type of exercise training does not induce the acute activation of muscle protein synthesis and chronic muscle hypertrophy even though mTORC1 is highly activated [2–4]. These facts indicate the possibility that an excessively shortened recovery influences the downstream targets of the mTORC1 pathway.

mTORC1 has two main downstream targets, p70S6K and 4E binding protein 1 (4EBP1), which are used as indicators of mTORC1 activity. 4EBP1 binds eukaryotic initiation factor 4E (eIF4E) and regulates translation initiation, and activation of mTORC1 promotes dissociation of 4EBP1 from eIF4E, a cap-binding protein [9]. The dissociated eIF4E binds to eIF4G, a scaffold protein, and forms the eIF4F complex with eIF4A. The formed eIF4F complex mediates the recruitment of the 43S preinitiation complex to the mRNA, promoting cap-dependent translation [10]. However, some types of stress can cause mTORC1-independent effects on eIF4F complex formation. In a septic condition characterized by systemic inflammation, leucine administration is reported to increase the phosphorylation of p70S6K but not the phosphorylation of 4EBP1, the dissociation of eIF4E from 4EBP1, or the association of eIF4E with eIF4G in rat skeletal muscle [11]. Another study reported that sepsis also inhibited the association of eIF4E with eIF4G, but the phosphorylation of p70S6K, phosphorylation of 4EBP1, and dissociation of eIF4E from 4EBP1 were unaffected in rat gastrocnemius muscle perfused with insulin [12]. Repetitive resistance exercise with insufficient recovery is also known to activate inflammation [2–4]. Therefore, this type of exercise training possibly impairs eIF4F complex formation, even though mTORC1 is highly activated, which may contribute to the attenuation of chronic muscle hypertrophy and/or acute activation of muscle protein synthesis.

To gain new insight into the mechanisms underlying the blunting of resistance training effects by insufficient recovery, we subjected the mice to resistance exercise every 72, 24, and 8 h and investigated the changes in eIF4E distribution and related factors after three successive bouts of resistance exercise. Resistance exercise training with 72 h of recovery is recommended and practically used in humans and known to activate effective protein synthesis in mouse skeletal muscle [3, 13]. Based on these facts, we used 72 h of recovery as the standard recovery period. Moreover, we used 24 h of recovery, which can be used in humans as a short recovery period, and 8 h of recovery, which is virtually impractical as it is too short of a recovery period. In our previous study, we observed that phosphorylation and activation of 4EBP1 after bouts of resistance exercise were not inhibited by an excessively shortened recovery [3]. Therefore, we hypothesized that excessive shortening of recovery attenuates the association of eIF4E with eIF4G.

## Materials and methods

### Animals and experimental design

Eighteen male C57BL/6J mice (10 weeks old, 22–25 g) were obtained from CLEA Japan (Tokyo, Japan). All animals were housed in an environment maintained at 22 ± 2 °C under a 12-h/12-h light–dark cycle and provided with food and water ad libitum. Mice were randomly divided into three groups, which were subjected to resistance exercise every 72 (72H, *n* = 6), 24 (24H, *n* = 6), and 8 h (8H, *n* = 6). All animals completed 3 sessions of resistance exercise for the right gastrocnemius muscle. Six hours after the last exercise session, mice were anesthetized and euthanatized by cervical dislocation, and muscle samples were collected. Three hours before the last exercise session, food was withdrawn, and the mice were fasted. To reduce the influence of fasting on the quality of recovery, especially in the 8H group, we shortened the fasting length from overnight, which was performed in our previous study, to 3 h before the last exercise session [3]. Collected muscles were frozen at  −  80 °C until use. This study was approved by the Ethics Committee for Animal Experiments at the University of Tokyo (27-13).

### Resistance exercise protocol

Resistance exercise was conducted according to the protocol described by Ogasawara et al. [14] with a slight modification. Briefly, under isoflurane anesthesia, the hair on the right hind limb of each mouse was shaved off, and the skin was cleaned with alcohol wipes. The right foot of each mouse was firmly attached to the footplate (the ankle joint angle was positioned at 90° relative to the tibia) in the prone position, electrodes (Vitrode V, Ag/AgCl; Nihon Kohden, Tokyo, Japan) were placed on both sides of the gastrocnemius muscle, and the muscle was stimulated percutaneously. Resistance exercise was performed by maximal isometric contractions (five sets of 3 s × 10 contractions with a 7-s interval between contractions and 3-min rest intervals). The left gastrocnemius muscle was unstimulated, and that in the 72H group served as the control. In a previous study, we contracted the tibialis anterior muscle via percutaneous stimulation on the deep peroneal nerve [3]. However, stimulating the muscle directly could eliminate the influence on the condition of the motor nerve, possibly caused by repeated electrical stimulation. Additionally, using the gastrocnemius muscle, we can obtain more samples compared with the tibialis anterior muscle. For these reasons, we used direct percutaneous electrical stimulation on the gastrocnemius muscle in the present study.

### Muscle protein synthesis

Muscle protein synthesis was measured according to the SUnSET method [15]. Under anesthesia, 0.04 μmol/g body weight puromycin diluted in phosphate-buffered saline was intraperitoneally injected into each mouse. The gastrocnemius muscles were removed 15 min after puromycin administration. Following homogenization, samples were centrifuged at 2000×*g* for 3 min at 4 °C, and the supernatant was collected and processed for western blotting using an anti-puromycin antibody (Cat # MABE343, Merck Millipore).

### Western blotting

For western blotting analysis, muscle samples were homogenized and analyzed as described previously [4]. Briefly, muscle samples were homogenized in RIPA buffer (Thermo Fisher Scientific, Waltham, MA, USA) containing Halt™ protease and phosphatase inhibitor cocktail (Thermo Fisher Scientific) and centrifuged at 10,000×*g* for 10 min at 4 °C. The supernatant was collected, and the protein concentration of each sample was determined using a protein concentration determination kit (DC™ Protein Assay kit, BioRad, USA). Samples were diluted in 3× Blue Loading Buffer [Cell Signaling Technology (CST), Danvers, MA, USA] and boiled at 95 °C for 5 min. Equal amounts of proteins were then subjected to 7.5%, 10%, or 12% TGX gel (BioRad) electrophoresis and subsequently transferred to polyvinylidene difluoride membranes. Membranes were blocked in 5% skim milk in Tris-buffered saline with Tween 20 (TBST) for 1 h at room temperature and subsequently incubated overnight at 4 °C with the following primary antibodies: p-p70S6K (Thr389, #9205; CST), p70S6K (#2708; CST), p-rpS6 (Ser240/244, #2215; CST), rpS6 (#2217; CST), p-4EBP1 (Thr37/46, #9459; CST), 4EBP1 (#9644; CST), eIF4E (#2067; CST), eIF4G (#2617; CST), eIF4A (#2013; CST), eIF4H (#3469; CST), p-eEF2 (Thr56, #2331; CST), eEF2 (#2332; CST), p-eIF4B (Ser422, #3591; CST), eIF4B (#3592, CST), p-eIF2α (Ser51, #3597; CST), and eIF2α (#5324; CST). Membranes were then incubated for 1 h at room temperature with the appropriate secondary antibodies and visualized using chemiluminescent reagents (Clarity™ Western ECL Substrate, BioRad). Bands were detected and quantified with ChemiDoc XRS (BioRad).

### Immunoprecipitation assays

Muscle samples were homogenized in lysis buffer containing 25 mM Tris-HCl (pH = 7.4), 50 mM NaCl, 1% Nonidet P-40, 1 mM EDTA, 5% glycerol, and cOmplete Mini protease inhibitor cocktail (Sigma-Aldrich, St. Louis, MO, USA). Immunoprecipitations were performed using the Dynabeads^®^ Protein A immunoprecipitation kit (Invitrogen, Carlsbad, CA, USA) according to the manufacturer’s instructions using an anti-eIF4E antibody (RN006M; Medical & Biological Laboratories, Japan). To confirm the validity, a negative control sample was prepared using a mouse IgG2a isotype control antibody (M076-3; Medical & Biological Laboratories, Japan). Bound protein was eluted in 3× Blue Loading Buffer (CST). The lysate was then processed by western blot analysis using anti-4EBP1 (#9644; CST), anti-eIF4G (#2617; CST), and anti-eIF4E (#2067; CST) antibodies.

### Statistical analysis

Data were analyzed using one-way ANOVA and post hoc analysis was performed using *t*-test with Benjamini–Hochberg false discovery rate correction for multiple comparisons when appropriate. All values are expressed as the mean ± standard error of the mean (SEM). Statistical significance was indicated by *P* < 0.05. Statistical analyses were performed using GraphPad Prism 8 (GraphPad Software, CA, USA).

## Results

### Muscle protein synthesis

**Fig. 1:**
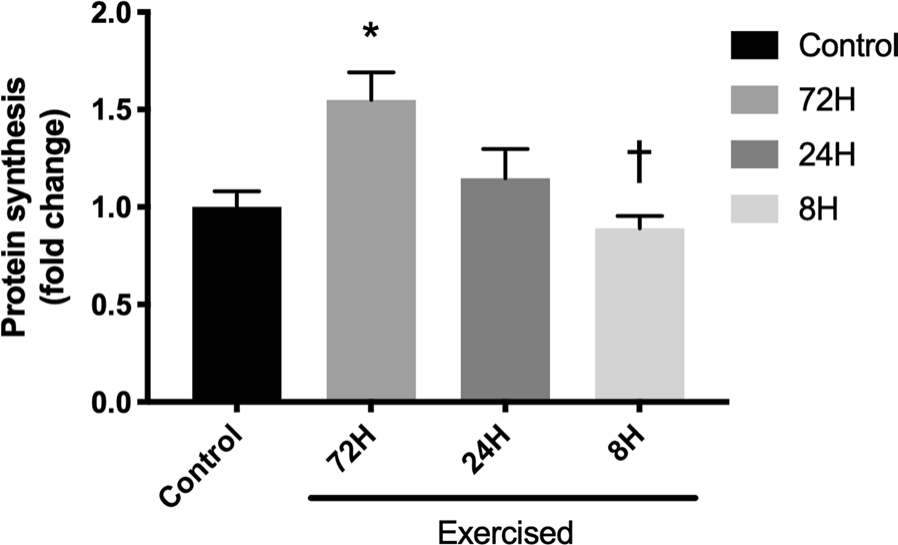
Muscle protein synthesis after three bouts of resistance exercise. Data are expressed relative to the Control as the mean ± SE. *P < 0.05 vs. Control, ^†^*P* < 0.05 vs. 72H group

Bouts of resistance exercise increased muscle protein synthesis in the 72H group (*P* = 0.004), but the response was decreased with shortening of recovery, and that in the 8H group was significantly lower than that in the 72H group (*P* = 0.021, Fig. 1).

### mTORC1 signaling

**Fig. 2:**
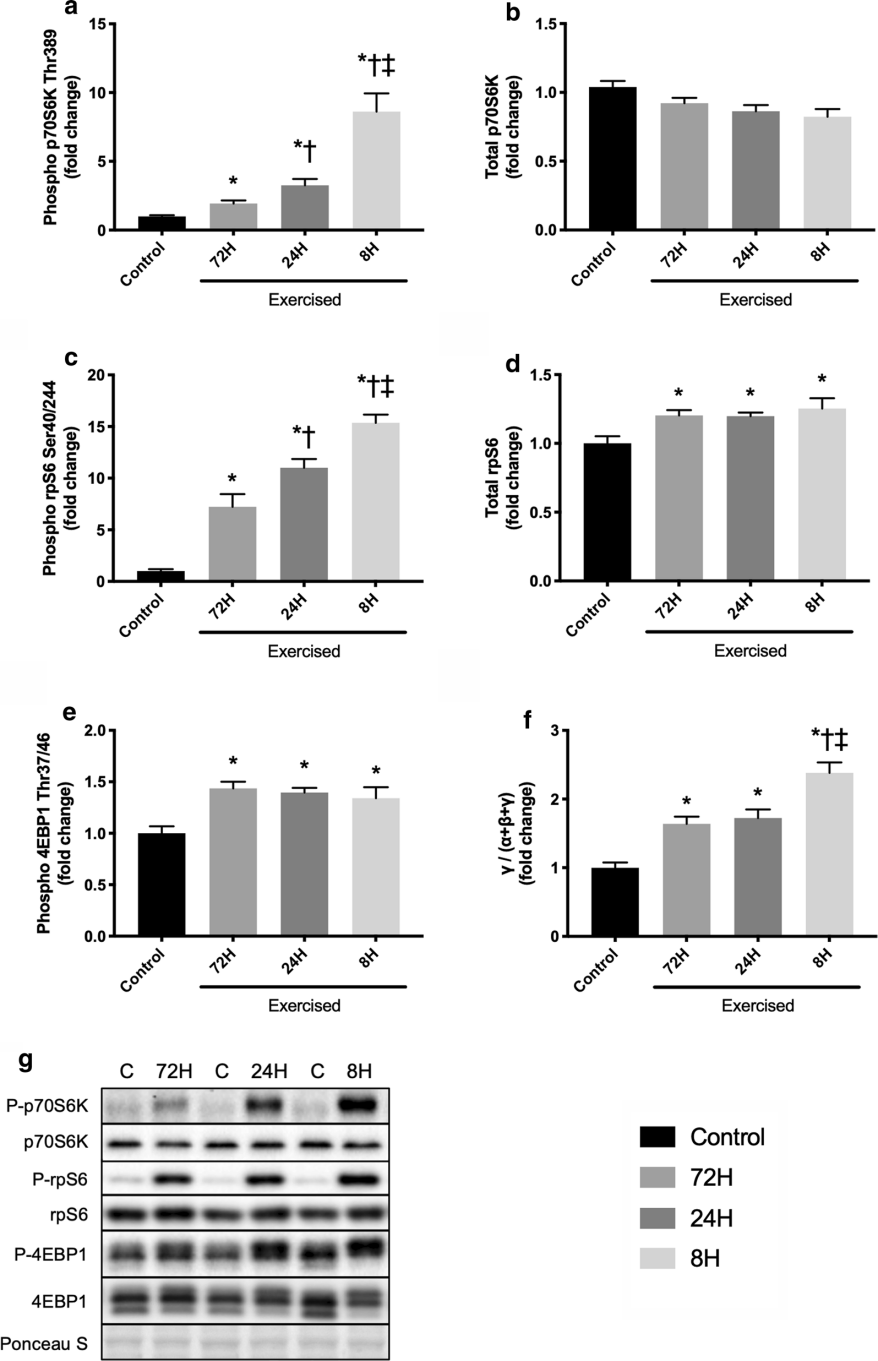
Protein expression of mTORC1 signaling-related factors after three bouts of resistance exercise. Protein expression of phosphorylated p70S6K (Thr389, **a**), p70S6K (**b**), phosphorylated rpS6 (Ser240/244, **c**), rpS6 (**d**), phosphorylated 4EBP1 (Thr37/46, **e**), 4EBP1 gamma form ratio (**f**), and representative bands (**g**). Data are expressed relative to the Control as the mean ± SE. *P < 0.05 vs. Control, ^†^*P* < 0.05 vs. 72H group, ^‡^*P* < 0.05 vs. 24H group

The phosphorylated form of p70S6K was increased in all exercise groups, with the magnitude increasing with shortening of recovery (Control vs. 72H, *P* = 0.004; Control vs. 24H, *P* = 0.002; Control vs. 8H, *P* = 0.001; 72H vs. 24H, *P* = 0.029; 72H vs. 8H, *P* = 0.002; 24H vs. 8H, *P* = 0.004; Fig. 2a). Similar results were observed for the phosphorylated form of rpS6 (Control vs. 72H, *P* = 0.001; Control vs. 24H, *P* < 0.001; Control vs. 8H, *P* < 0.001; 72H vs. 24H, *P* = 0.031; 72H vs. 8H, *P* = 0.001; 24H vs. 8H, *P* = 0.005; Fig. 2c). Total p70S6K expression was not changed by exercise (Control vs. 72H, *P* = 0.1381; Control vs. 24H, *P* = 0.051; Control vs. 8H, *P* = 0.075; 72H vs. 24H, *P* = 0.407; 72H vs. 8H, *P* = 0.268; 24H vs. 8H, *P* = 0.590; Fig. 2b), whereas total rpS6 expression was increased in all exercise groups (Control vs. 72H, *P* = 0.032; Control vs. 24H, *P* = 0.032; Control vs. 8H, *P* = 0.047), but the degree was not different among the groups (72H vs. 24H, *P* = 0.918; 72H vs. 8H, *P* = 0.661; 24H vs. 8H, *P* = 0.715; Fig. 2d). The phosphorylated form of 4EBP1 was increased in all exercise groups compared with the control (Control vs. 72H, *P* = 0.003; Control vs. 24H, *P* = 0.003; Control vs. 8H, *P* = 0.046), and no significant differences were observed between any of the exercise groups (72H vs. 24H, *P* = 0.515; 72H vs. 8H, *P* = 0.695; 24H vs. 8H, *P* = 0.649; Fig. 2e). The active form ratio (*γ* form ratio), a marker of 4EBP1 activity, was increased in all exercise groups (Control vs. 72H, *P* = 0.001; Control vs. 24H, *P* = 0.001; Control vs. 8H, *P* < 0.001), and the highest ratio was observed in the 8H group (72H vs. 8H, *P* = 0.004; 24H vs. 8H, *P* = 0.009; Fig. 2f).

### Expression of eIF4F components

**Fig. 3:**
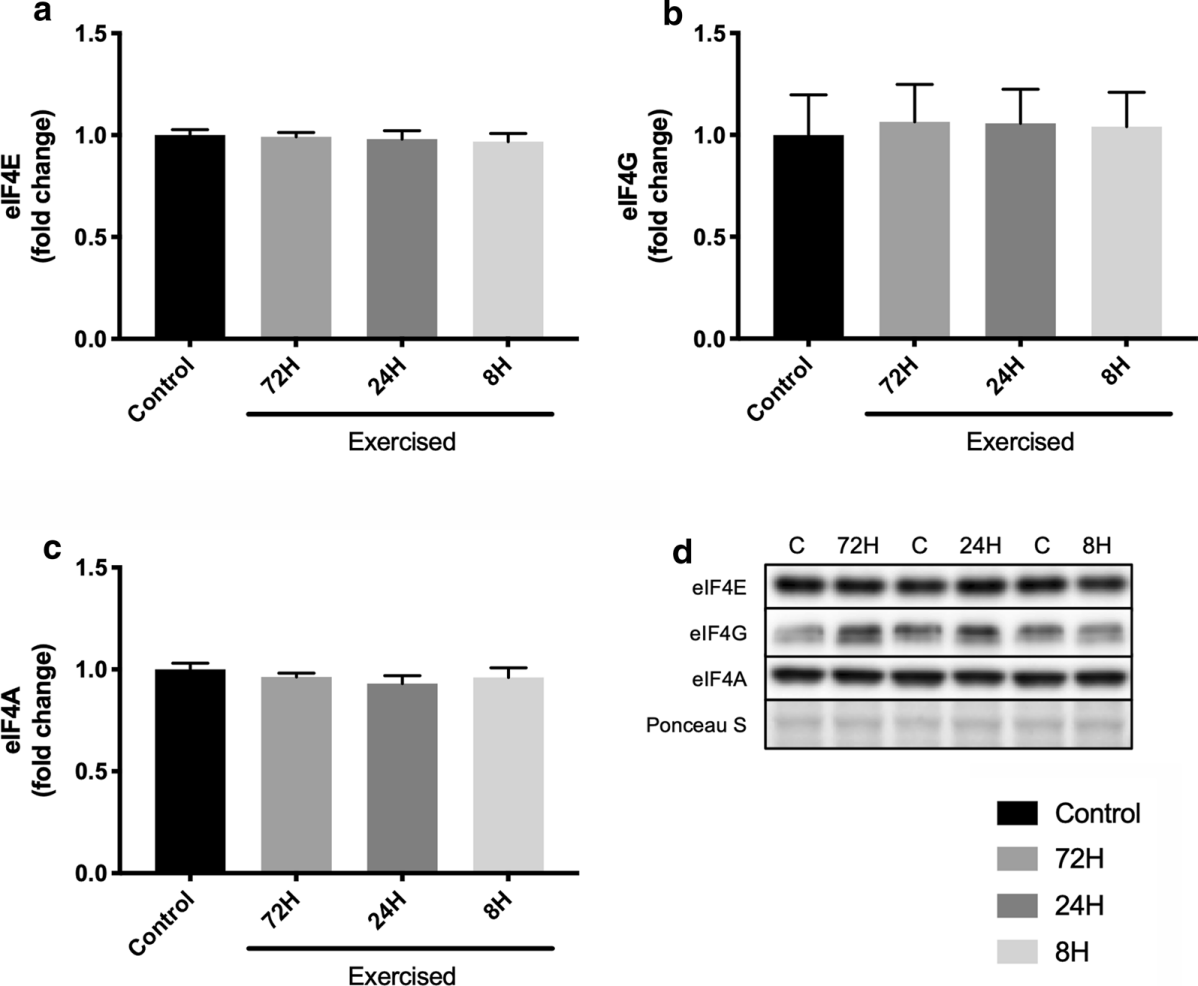
Protein expression of eIF4F components after three bouts of resistance exercise. Protein expression of eIF4E (**a**), eIF4G (**b**), eIF4A (**c**), and representative bands (**d**). Data are expressed relative to the Control as the mean ± SE

To confirm the effect of shortening recovery on the expression of eIF4F components, we measured the protein expression of eIF4E, eIF4G, and eIF4A, which are components of the eIF4F complex. As shown in Fig. 3, no significant differences in protein expression were observed between any of the groups (one-way ANOVA *P* = 0.912, 0.994, and 0.602 for eIF4E, eIF4G, and eIF4A, respectively; Fig. 3a–c).

### eIF4E distribution

**Fig. 4:**
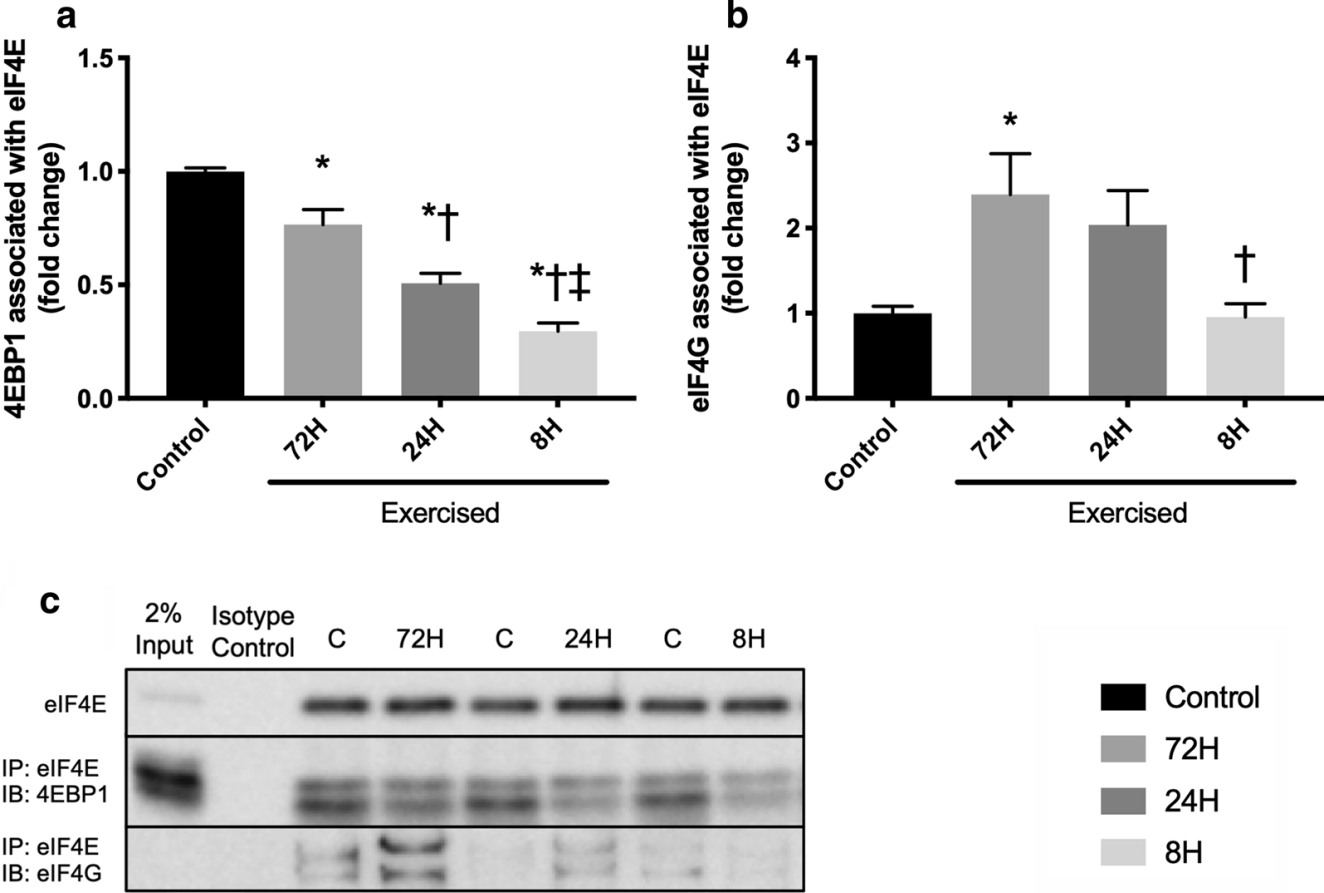
Distribution of eIF4E after three bouts of resistance exercise. Protein expression of 4EBP1 associated with eIF4E (**a**), eIF4G associated with eIF4E (**b**), and representative bands (**c**). Data are expressed relative to the Control as the mean ± SE. *P < 0.05 vs. Control, ^†^*P* < 0.05 vs. 72H group, ^‡^*P* < 0.05 vs. 24H group

We next investigated alterations in eIF4E distribution to examine the influence of shortening recovery on regulatory steps in translational control. Bouts of resistance exercise decreased the expression of 4EBP1 associated with eIF4E, with the magnitude increasing with the shortening of recovery (Control vs. 72H, *P* = 0.008; Control vs. 24H, *P* < 0.001; Control vs. 8H, *P* < 0.001; 72H vs. 24H, *P* = 0.009; 72H vs. 8H, *P* < 0.001; 24H vs. 8H, *P* = 0.006; Fig. 4a). In contrast, bouts of resistance exercise increased the expression of eIF4G associated with eIF4E in the 72H group (*P* = 0.048), but the magnitude was decreased with shortening of recovery, and that in the 8H group was significantly lower than that in the 72H recovery group (*P* = 0.048, Fig. 4b).

### eIF4B and eIF4H

**Fig. 5:**
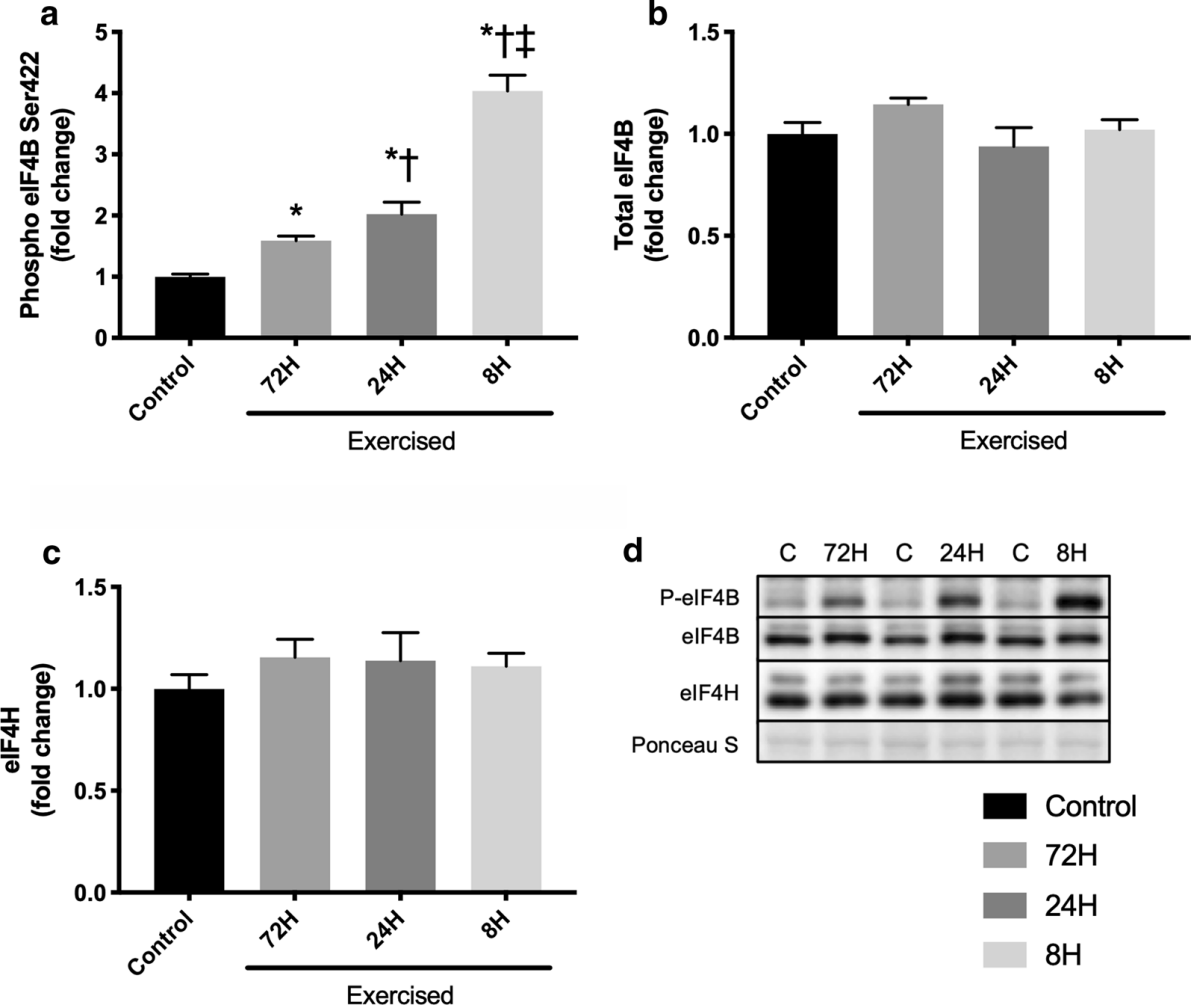
Expression of accessory proteins for eIF4A. Protein expression of phosphorylated eIF4B (Ser422, **a**), eIF4B (**b**), eIF4H (**c**), and representative bands (**d**). Data are expressed relative to the Control as the mean ± SE. *P < 0.05 vs. Control, ^†^*P* < 0.05 vs. 72H group, ^‡^*P* < 0.05 vs. 24H group

We additionally investigated the expression of eIF4B and eIF4H, accessory proteins for eIF4A. Bouts of resistance exercise increased phosphorylated eIF4B expression, with the magnitude increasing with the shortening of recovery (Control vs. 72H, *P* < 0.001; Control vs. 24H, *P* < 0.001; Control vs. 8H, *P* < 0.001; 72H vs. 24H, *P* = 0.064; 72H vs. 8H, *P* < 0.001; 24H vs. 8H, *P* < 0.001; Fig. 5a). No significant differences in total protein expression of eIF4B and eIF4H were observed between any of the groups (one-way ANOVA *P* = 0.149 and 0.664, respectively; Fig. 5b, c).

### Other translation-controlling factors

**Fig. 6:**
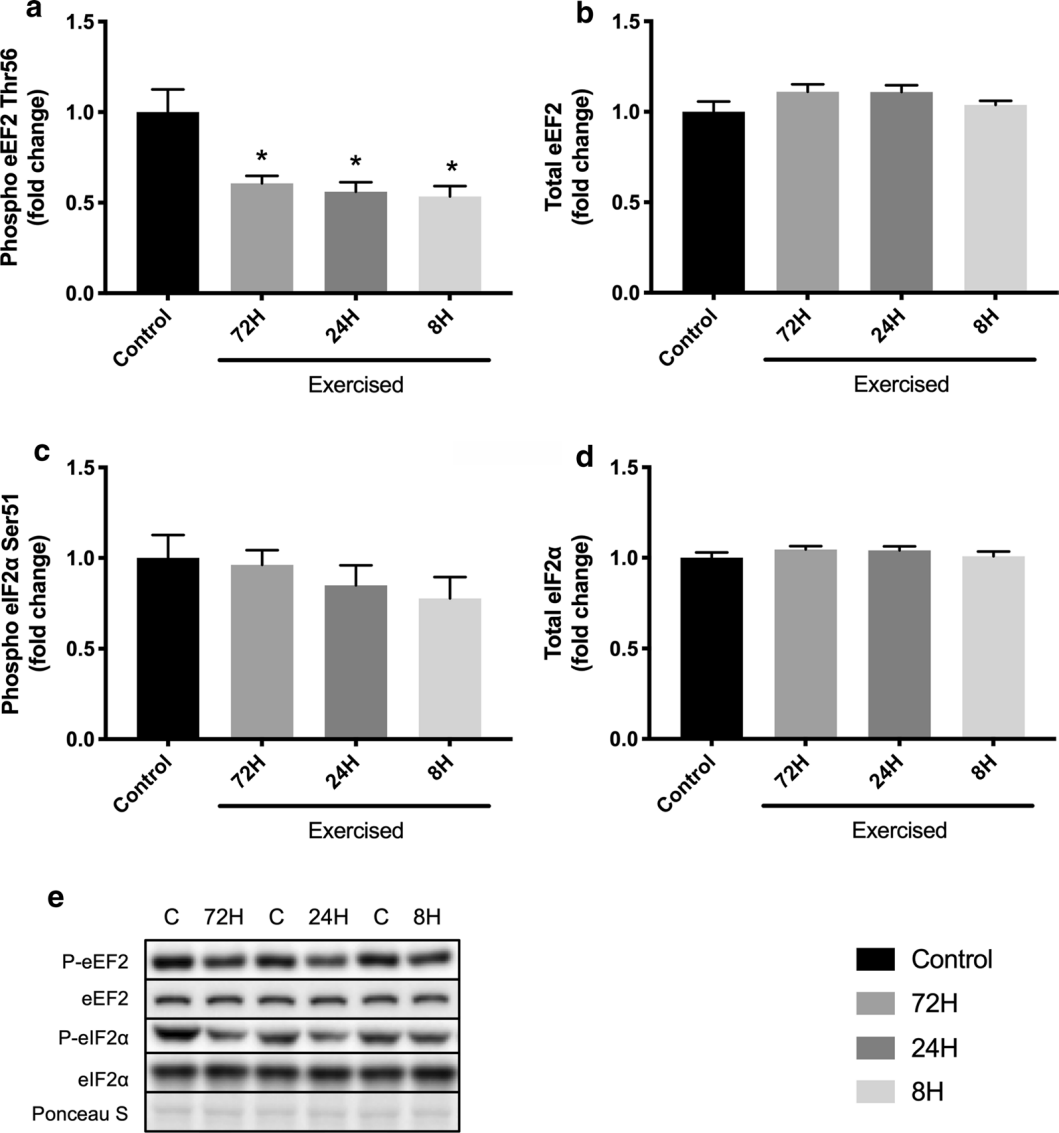
Expressions of eEF2 and eIF2α. Protein expression of phosphorylated eEF2 (Thr56, **a**), eEF2 (**b**), phosphorylated eIF2α (Ser51, **c**), eIF2α (**d**), and representative bands (**e**). Data are expressed relative to the Control as the mean ± SE. *P < 0.05 vs. Control

Finally, we investigated eEF2 and eIF2α, which are factors. Bouts of resistance exercise decreased phosphorylated eEF2 expression, which was unaffected by the duration of recovery time (Control vs. 72H, *P* = 0.028; Control vs. 24H, *P* = 0.027; Control vs. 8H, *P* = 0.027; 72H vs. 24H, *P* = 0.608; 72H vs. 8H, *P* = 0.493; 24H vs. 8H, *P* = 0.732; Fig. 6a). The levels of total eEF2 and both phosphorylated and total eIF2α were not changed by bouts of resistance exercise or shortening of recovery (one-way ANOVA *P* = 0.185, 0.478, and 0.481, respectively; Fig. 6b–d).

## Discussion

The present study investigated the effects of shortening recovery between bouts of resistance exercise on translation-controlling factors. The main findings of this study are as follows: (1) shortening of recovery further promoted dissociation of eIF4E from 4EBP1 after bouts of resistance exercise, but the association of eIF4E with eIF4G was decreased, (2) three successive bouts of resistance exercise did not change the expression of eIF4F components regardless of the duration of recovery, and (3) similar to the previous study, three successive bouts of resistance exercise with 8-h recovery activated mTORC1 to a greater extent than with 72-h recovery but did not increase protein synthesis. These results suggest that excessive shortening of recovery between exercise bouts inhibits the formation of eIF4F even though mTORC1 is highly activated, which may be involved in the attenuation of protein synthesis activation after bouts of resistance exercise.

Three successive bouts of resistance exercise with 72-h recovery increased the association of eIF4E with eIF4G in the present study. However, in shorter recovery groups, bouts of resistance exercise did not increase the association of eIF4E to eIF4G. In previous studies, the association of eIF4E with eIF4G and protein synthesis showed similar changes in physiological conditions [16–20]. Considering that the expression of eIF4F components did not change among groups, our results suggest that shortening of recovery inhibited the formation of eIF4F, resulting in attenuation of protein synthesis activation. In contrast, shorter recovery groups activated mTORC1 to a greater extent and promoted dissociation of eIF4E and 4EBP1 more than the 72-h recovery group in the present study. Previous studies reported corresponding changes between mTORC1 activity and association of eIF4E with eIF4G [21–25]. However, as mentioned above, some studies reported mTORC1-independent inhibition of eIF4F formation in vivo and in vitro [11–13]. Although the detailed mechanisms regulating eIF4F complex formation remain unclear, our results provide evidence that mTORC1 does not dominantly regulate the eIF4F complex. Elucidating the underlying mechanisms would contribute to the development of a new method to enhance the efficiency of resistance exercise training.

eIF4A is a DEAD-box RNA helicase that unwinds the secondary structure of mRNA [26]. eIF4B and eIF4H potentially enhance the translation rate through modulation of helicase activity of eIF4A [27, 28]. The present study reported that the expression of phosphorylated eIF4B after bouts of resistance exercise increased with shortening of recovery. eIF4B is known to be phosphorylated by p70S6K [29], a downstream target of mTORC1, and a previous ex vivo study reported that muscle contraction not only activated mTORC1 but also phosphorylated eIF4B [30]. These findings lead us to predict that the greater increase in phosphorylated eIF4B expression observed in shorter recovery groups likely results from increased phosphorylation by p70S6K. In contrast, the expression of eIF4H was not changed by bouts of resistance exercise and shortening of recovery in the present study. To our knowledge, changes in eIF4H by physiological anabolic stimulus have not been reported. A previous study observed no significant changes in eIF4H expression in synergist ablated mouse extensor digitorum longus muscle [31]. These results suggest that mechanical stress does not affect the expression of eIF4H. However, the status of eIF4B and eIF4H likely did not affect the inactivation of muscle protein synthesis after bouts of resistance exercise caused by excessive shortening of recovery.

To explore other factors involved in translation, we investigated eEF2 and eIF2α. Resistance exercise dephosphorylates eEF2, which is thought to promote translation [32]. eEF2 is regulated by its kinase, eEF2K. eEF2K is a Ca/calmodulin-dependent kinase that is known to be inactivated by mTORC, p70S6K, and others [33]. In the present study, shorter recovery groups activated mTORC1 to a greater extent than the 72-h recovery group. Therefore, eEF2 was expected to be mainly dephosphorylated in shorter recovery groups. Surprisingly, three successive bouts of resistance exercise decreased the expression of phosphorylated eEF2, but the magnitude was not affected by shortening of recovery. eEF2 is phosphorylated through eEF2K by AMP-activated protein kinase (AMPK) and oxidative stress, which are known to be activated or increased by repetitive resistance exercise with shorter recovery [2, 3, 34–36]. These facts may indicate that dephosphorylation of eEF2 induced by mTORC was offset by activation or increase of AMPK and oxidative stress in shorter recovery groups. Meanwhile, a previous study reported that the dephosphorylation of eEF2 induced by resistance exercise was inhibited by the ATP-competitive mTOR kinase inhibitor AZD8055 but not rapamycin in rats [7]. These facts also suggest that repetitive resistance exercise with shorter recovery did not enhance the activation of rapamycin-insensitive mTORC, resulting in modest dephosphorylation of eEF2. To elucidate these mechanisms, further investigations are required. eIF2α is activated by various cellular stress (e.g., oxidative stress, endoplasmic reticulum stress) and downregulates translation. A previous study reported that unaccustomed resistance exercise increases its mRNA expression in human skeletal muscle [37]. However, consistent with our previous study [3], bouts of resistance exercise did not change phosphorylated eIF2α expression despite changing the recovery duration. These observations lead us to conclude that eEF2 and eIF2α are not involved in the suppression of protein synthesis activation caused by excessively short recovery periods.

In agreement with a previous study, shorter recovery groups activated mTORC1 to a greater extent but did not activate protein synthesis [3]. However, the present study did not show significant increase of protein synthesis in the 24-h recovery group, in contrast to the previous study. As mentioned above, the experimental design of the present study was different from the previous study, which possibly influenced the responses. Fasting is known to reduce muscle protein synthesis [15]. In comparison with our previous study, the fasting period was shortened from overnight to 3 h [3]. Shortening of the fasting period would attenuate the reduction in basal muscle protein synthesis, which possibly made it more difficult to detect the activation of muscle protein synthesis after bouts of resistance exercise in shorter recovery groups. Additionally, the gastrocnemius muscle is one of the agonist muscles for walking in mice [38]. Although the tibialis anterior muscle is also used in walking, there is a difference in load for each muscle. Activity levels were not measured in the present study, and no significant difference in protein synthesis was observed between control legs among groups (data had not shown). However, mice in shorter recovery groups possibly avoided using exercised legs because of muscle soreness, which might attenuate muscle protein synthesis in exercised legs and contribute to the differences between our previous and present study. Therefore, shortening of the fasting period and using the gastrocnemius muscle could cause differences in the response to various recovery times after bouts of resistance exercise.

## Conclusion

Our results indicate that repeated bouts of resistance exercise with conventional recovery activated mTORC1, dissociated eIF4E and 4EBP1, increased the association of eIF4E and eIF4G, and increased muscle protein synthesis. In contrast, shortening of recovery augmented the activation of mTORC1 and the dissociation of eIF4E and 4EBP1 but suppressed the increase in the association between eIF4E and eIF4G and muscle protein synthesis after bouts of resistance exercise. Considering that the expression or phosphorylation of eIF4B, eIF4H, eEF2, and eIF2α were not negatively affected by shortening of recovery, the reduced association of eIF4E and eIF4G was possibly involved in attenuation of resistance exercise training effects caused by shortening of recovery. These findings contribute to a better understanding of the mechanisms involved in the blunting of resistance training effects by insufficient recovery. Further studies are required to clarify the mechanism underlying the dissociation of eIF4E and eIF4G caused by excessive shortening of recovery after bouts of resistance exercise. Additionally, investigating the responses in humans would be useful for clinical application in the future.

## Data Availability

The data that support the findings of this study are available from the corresponding author on reasonable request.

## References

[1] Coffey VG, Reeder DW, Lancaster GI, Yeo WK, Febbraio MA, Yaspelkis BB, Hawley JA (2007). Effect of high-frequency resistance exercise on adaptive responses in skeletal muscle. Med Sci Sports Exerc.

[2] Takegaki J, Ogasawara R, Tamura Y, Takagi R, Arihara Y, Tsutaki A, Nakazato K, Ishii N (2017). Repeated bouts of resistance exercise with short recovery periods activates mTOR signaling, but not protein synthesis, in mouse skeletal muscle. Physiol Rep.

[3] Takegaki J, Ogasawara R, Kotani T, Tamura Y, Takagi R, Nakazato K, Ishii N (2019). Influence of shortened recovery between resistance exercise sessions on muscle-hypertrophic effect in rat skeletal muscle. Physiol Rep.

[4] Kraemer WJ, Ratamess N (2004). Fundamentals of resistance training: progression and exercise prescription. Med Sci Sports Exerc.

[5] West DW, Baehr LM, Marcotte GR, Chason CM, Tolento L, Gomes AV, Bodine SC, Baar K (2016). Acute resistance exercise activates rapamycin-sensitive and-insensitive mechanisms that control translational activity and capacity in skeletal muscle. J Physiol.

[6] Ogasawara R, Fujita S, Hornberger TA, Kitaoka Y, Makanae Y, Nakazato K, Ishii N (2016). The role of mTOR signalling in the regulation of skeletal muscle mass in a rodent model of resistance exercise. Sci Rep.

[7] Ogasawara R, Suginohara T (2018). Rapamycin-insensitive mechanistic target of rapamycin regulates basal and resistance exercise-induced muscle protein synthesis. FASEB J.

[8] You JS, McNally RM, Jacobs BL, Privett RE, Gundermann DM, Lin KH, Steinert ND, Goodman CA, Hornberger TA (2018). The role of raptor in the mechanical load-induced regulation of mTOR signaling, protein synthesis, and skeletal muscle hypertrophy. FASEB J.

[9] Haghighat A, Mader S, Pause A, Sonenberg N (1995). Repression of cap-dependent translation by 4E-binding protein 1: competition with p220 for binding to eukaryotic initiation factor-4E. EMBO J.

[10] Pain VM (1996). Initiation of protein synthesis in eukaryotic cells. Euro J Biochem.

[11] Lang CH, Frost RA (2005). Endotoxin disrupts the leucine-signaling pathway involving phosphorylation of mTOR, 4E-BP1, and S6K1 in skeletal muscle. J Cell Physiol.

[12] Vary TC, Jefferson LS, Kimball SR (2001). Insulin fails to stimulate muscle protein synthesis in sepsis despite unimpaired signaling to 4E-BP1 and S6K1. Am J Physiol Endocrinol Metab.

[13] Ratamess NA, Alvar BA, Evetoch TK, Housh TJ, Kibler WB, Kraemer WJ, Triplett NT (2009). Progression models in resistance training for healthy adults. Med Sci Sports Exerc.

[14] Ogasawara R, Sato K, Matsutani K, Nakazato K, Fujita S (2014). The order of concurrent endurance and resistance exercise modifies mTOR signaling and protein synthesis in rat skeletal muscle. Am J Physiol Endocrinol Metab.

[15] Goodman CA, Mabrey DM, Frey JW, Miu MH, Schmidt EK, Pierre P, Hornberger TA (2011). Novel insights into the regulation of skeletal muscle protein synthesis as revealed by a new nonradioactive in vivo technique. FASEB J.

[16] Gautsch T, Anthony J, Kimball S, Paul G, Layman D, Jefferson L (1998). Availability of eIF4E regulates skeletal muscle protein synthesis during recovery from exercise. Am J Physiol Cell Physiol.

[17] Vary TC, Jefferson LS, Kimball SR (1999). Amino acid-induced stimulation of translation initiation in rat skeletal muscle. Am J Physiol Endocrinol Metab.

[18] Lang CH, Frost RA, Jefferson LS, Kimball SR, Vary TC (2000). Endotoxin-induced decrease in muscle protein synthesis is associated with changes in eIF2B, eIF4E, and IGF-I. Am J Physiol Endocrinol Metab.

[19] Bolster DR, Crozier SJ, Kimball SR, Jefferson LS (2002). AMP-activated protein kinase suppresses protein synthesis in rat skeletal muscle through down-regulated mammalian target of rapamycin (mTOR) signaling. J Biol Chem.

[20] Gazzaneo MC, Suryawan A, Orellana RA, Torrazza RM, El-Kadi SW, Wilson FA, Kimball SR, Srivastava N, Nguyen HV, Fiorotto ML (2011). Intermittent bolus feeding has a greater stimulatory effect on protein synthesis in skeletal muscle than continuous feeding in neonatal pigs. J Nutr.

[21] Crozier SJ, Kimball SR, Emmert SW, Anthony JC, Jefferson LS (2005). Oral leucine administration stimulates protein synthesis in rat skeletal muscle. J Nutr.

[22] Wilson FA, Suryawan A, Orellana RA, Kimball SR, Gazzaneo MC, Nguyen HV, Fiorotto ML, Davis TA (2009). Feeding rapidly stimulates protein synthesis in skeletal muscle of neonatal pigs by enhancing translation initiation. J Nutr.

[23] Kumar V, Frost RA, Lang CH (2002). Alcohol impairs insulin and IGF-I stimulation of S6K1 but not 4E-BP1 in skeletal muscle. Am J Physiol Endocrinol Metab.

[24] Lang CH, Frost RA, Deshpande N, Kumar V, Vary TC, Jefferson LS, Kimball SR (2003). Alcohol impairs leucine-mediated phosphorylation of 4E-BP1, S6K1, eIF4G, and mTOR in skeletal muscle. Am J Physiol Endocrinol Metab.

[25] Lang CH, Frost RA (2006). Glucocorticoids and TNFα interact cooperatively to mediate sepsis-induced leucine resistance in skeletal muscle. Mol Med.

[26] Lu WT, Wilczynska A, Smith E, Bushell M (2014). The diverse roles of the eIF4A family: you are the company you keep. Biochem Soc Trans.

[27] Richter-Cook NJ, Dever TE, Hensold JO, Merrick WC (1998). Purification and characterization of a new eukaryotic protein translation factor. Eukaryotic initiation factor 4H. J Biol Chem.

[28] Rogers GW, Richter NJ, Lima WF, Merrick WC (2001). Modulation of the helicase activity of eIF4A by eIF4B, eIF4H, and eIF4F. J Biol Chem.

[29] Raught B, Peiretti F, Gingras AC, Livingstone M, Shahbazian D, Mayeur GL, Polakiewicz RD, Sonenberg N, Hershey JW (2004). Phosphorylation of eukaryotic translation initiation factor 4B Ser422 is modulated by S6 kinases. EMBO J.

[30] Liu Y, Vertommen D, Rider MH, Lai YC (2013). Mammalian target of rapamycin-independent S6K1 and 4E-BP1 phosphorylation during contraction in rat skeletal muscle. Cell Signal.

[31] Pereira MG, Dyar KA, Nogara L, Solagna F, Marabita M, Baraldo M, Chemello F, Germinario E, Romanello V, Nolte H (2017). Comparative analysis of muscle hypertrophy models reveals divergent gene transcription profiles and points to translational regulation of muscle growth through increased mTOR signaling. Front Physiol.

[32] Dreyer HC, Fujita S, Cadenas JG, Chinkes DL, Volpi E, Rasmussen BB (2006). Resistance exercise increases AMPK activity and reduces 4E-BP1 phosphorylation and protein synthesis in human skeletal muscle. J Physiol.

[33] Browne GJ, Proud CG (2004). A novel mTOR-regulated phosphorylation site in elongation factor 2 kinase modulates the activity of the kinase and its binding to calmodulin. Mol Cell Biol.

[34] Horman S, Browne GJ, Krause U, Patel JV, Vertommen D, Bertrand L, Lavoinne A, Hue L, Proud CG, Rider MH (2002). Activation of AMP-activated protein kinase leads to the phosphorylation of elongation factor 2 and an inhibition of protein synthesis. Curr Biol.

[35] Sanchez M, Lin Y, Yang CC, McQuary P, Campos AR, Blanc PA, Wolf DA (2019). Cross talk between eIF2α and eEF2 phosphorylation pathways optimizes translational arrest in response to oxidative stress. Science.

[36] Thomson DM, Fick CA, Gordon SE (2008). AMPK activation attenuates S6K1, 4E-BP1, and eEF2 signaling responses to high-frequency electrically stimulated skeletal muscle contractions. J Appl Physiol.

[37] Ogborn DI, McKay BR, Crane JD, Parise G, Tarnopolsky MA (2014). The unfolded protein response is triggered following a single, unaccustomed resistance-exercise bout. Am J Physiol Regul Integr Comp Physiol.

[38] Pearson K, Acharya H, Fouad K (2005). A new electrode configuration for recording electromyographic activity in behaving mice. J Neurosci Meth.

